# Co-Ordination in Local Units

**Published:** 1921-08-13

**Authors:** 


### The Hospital

The Workers' Newspaper of Administrative Medicine and Institutional Life, Administration, National Insurance and Health.

No. 1836, Vol. LXX. SATURDAY, AUGUST 13, 1921. PRICE TWOPENCE

### CO-ORDINATION IN LOCAL UNITS.

Now that the report of Lord Cave's Committee has focused public attention upon the voluntary system as a whole, the peculiar problems of the London, the English, and the Scottish hospitals are being distinguished as they have never been before. One of the points often made is that the Scottish hospitals seem to be financially sounder than those in other parts of the country. Local pride is naturally flattered, but the gratification felt does not lead to an examination of the reasons which place the Scottish hospitals in this favourable light. The comparison stops at finance. A true comparison would go further, and would show that the Scottish hospitals have adopted a policy different from that pursued in the South. They have managed to finance themselves because they proceed very cautiously. Consequently the problem in Scotland is not so much to find more money for deficits or present needs as to provide more beds; the shortage is less of immediate finance than of future accommodation. If their accounts compare favourably with those of English hospitals the waiting-lists do not. The waiting-lists of Scottish hospitals are far too long.

We can thus distinguish the problem in London, the problem in the provinces, and the problem in Scotland. Co-operation in suitable areas is what is required. London has King Edward's Hospital Fund; the provinces at present have only the regional committees of the British Hospitals Association. Scotland, which has also its regional committee, needs in addition a centre of unity for hospitals outside Edinburgh and Glasgow. It does not greatly matter, perhaps, what the centre of unity is, but an essential preliminary is the establishment not merely of a uniform system of accounts but of the same financial year for all hospitals. Each centre of unity has to decide first which are the best means of raising money, and to organise its members accordingly. When that step has been taken, others toward saving expense, the prevention of overlapping, the most economical use of beds, particularly in Scotland, must follow. Competition has fostered one technique of administration. Co-operation must beget another. So soon as men have begun to be trained in an atmosphere of the latter several of the difficulties which seem so great to the elder generation will disappear. South of the Tweed energies must be directed to increasing the amount of income; north of the Tweed to increasing the number of beds.

Lord Cave's Committee has done valuable work in surveying the whole problem, and defining its different aspects in different parts of the country. The need is now for the different areas to form their appropriate organisations, and it is the formation of these which will also enable the local committees to secure the help of the Commission. For the recommendations of the local committees will fail of their effect unless they can point to an organisation in being to raise the quota from voluntary sources which is virtually made the qualification for receiving a grant at all.

